# Concept of cross border policy in public health: what is it and how does it

**DOI:** 10.1093/eurpub/ckac129.217

**Published:** 2022-10-25

**Authors:** B Van der Zanden

**Affiliations:** Foundation euPrevent, Heerlen, Netherlands

## Abstract

Cross-border cooperation depends on many factors. Not least because of the perception of a border and its influence. Literature shows that the perception of a border is different for different stakeholders. A citizen has a different perception of a border than a policy worker of the government. This influences the effects of cross-border policies. The first three presentations already show this indirectly. Apart from the perception of a border, it is important to look at how cross-border policy in the field of public health is actually made in Europe. On the basis of European policy documents, it has been analysed how policy and policy issues concerning cross-border public health care in Europe are established and what can be deduced from this for policy in cross-border regions such as the EMR. Elements in this are the ‘existence’, ‘genesis’, ‘influencers’ (+/- stakeholders) of cross-border policy and the potential relation with public health. Questions that will be addressed during the presentation are: What does cross-border policy look like? Is this also available in the field of public health? When yes how has this come about (genesis)? How is this concept of cross-border policy influencing the current way of looking at cross-border public health? Are there currently main ‘influencers’ that have impact on creating cross-border public health policy?

